# Complete Genome Sequence of *Serratia marcescens* U36365, a Green Pigment–Producing Strain Isolated from a Patient with Urinary Tract Infection

**DOI:** 10.1128/genomeA.00837-16

**Published:** 2016-08-11

**Authors:** Rani Diana Sahni, Rabecca Amalanathan, Naveen Kumar Devanga Ragupathi, John Mathai, Balaji Veeraraghavan, Indranil Biswas

**Affiliations:** a Department of Clinical Microbiology, Christian Medical College, Vellore, Tamil Nadu, India; b Department of Pediatric Surgery, Christian Medical College, Vellore, Tamil Nadu, India; c Department of Microbiology, Molecular Genetics and Immunology, University of Kansas Medical Center, Kansas City, Kansas, USA

## Abstract

*Serratia marcescens* is an emerging nosocomial pathogen associated with urinary and respiratory tract infections. In this study, we determined the genome of a green pigment–producing clinical strain, U36365, isolated from a hospital in Southern India. *De novo* assembly of PacBio long-read sequencing indicates that the U36365 genome consists of a chromosome of 5.12 Mbps and no plasmids.

## GENOME ANNOUNCEMENT

*Serratia marcescens* is an opportunistic nosocomial pathogen that causes occasional infections of the bloodstream, skin, and respiratory and urinary tracts (1). This organism is known for its production of a bright red pigment known as prodigiosin (2, 3). Treatment of *S. marcescens* infections can be challenging due to intrinsic and acquired resistance to numerous antibiotics (1, 4).

We have isolated *S. marcescens* strain U36365 from a 7-year-old male patient with a neurogenic bladder from Christian Medical College, Vellore, Tamil Nadu, India. The isolate was resistant to amoxicillin-clavulanic acid, cepodoxime, colistin, and nitrofurantoin. To mention, *S. marcescens* was known to be intrinsically resistant to nitrofurantoin, amoxicillin-clavulanic acid, and colistin. However, the isolate was susceptible to other commonly used antibiotics. This isolate produces a weakly β-hemolytic colony on a blood agar plate. Surprisingly, unlike other *S. marcescens* strains, U36365 produces a distinct green-pigmented colony on solid nutrient medium at 37°C.

To facilitate molecular studies, we determined the complete genome sequence of *S. marcescens* U36365 using PacBio single-molecule real-time (SMRT) technology (5). We prepared a 5- to 20-kb genomic DNA library suitable for P6/C4 chemistry. Using one SMRT cell on the PacBio RSII sequencing platform, we obtained 175,837 reads with a mean read length of 9.4 kb. The reads were assembled *de novo* with the Hierarchical Genome Assembly Process version 3 (HGAP3) (6) within SMRTAnalysis version 2.3.0 software. The best assembly was selected, and Minimus 2 was used for trimming the circular contig (7). SMRTAnalysis with the default parameters was used for detecting base modifications in the genome.

The assembled U36365 genome consists of a single circular chromosome with 5,125,866 bp containing 59.8% GC. The genome was annotated using BASys (8) and the Analysis Engine at the University of Maryland (9). The genome predicts 5,115 open reading frames, 22 rRNA genes, 89 tRNA genes, and one tmRNA gene. Three motifs with m6-adenosine and one motif with m4-cytosine methylations were detected in the genome. ResFinder-2.1 analysis at the CGE server returned no common resistance genes in the UA36365 genome. IslandViewer3 (10) analysis suggests that the U36365 genome harbors between five and 11 genomic islands. ISFinder analysis (11) indicates that the genome contains at least three Tn3 family of IS elements. Similarly, PHAST analysis predicts two complete prophage sequences in the genome (12). However, no clustered regularly interspaced short palindromic repeat (CRISPR) arrays were detected by CRISPRFinder (13). Finally, analysis by antiSMASH suggests seven putative secondary metabolite gene clusters for enterobactin, microcin, prodigiosin, ravidomycin, vanchrobactin, and xantholipin biosynthesis (14). The complete genome sequence of U36365 will be valuable for comparative genomic studies and for understanding biosynthesis of various secondary metabolites.

### Accession number(s).

The *S. marcescens* U36365 genome sequence was deposited in GenBank under the accession number CP016032. The version described in this manuscript is version CP016032.1.
